# Insulin Resistance as an Underlying Cause of Type 2 Diabetes in Adults Born with Low Birth Weight: A Narrative Review

**DOI:** 10.2174/0115733998390970251115094927

**Published:** 2026-01-09

**Authors:** Sana Ahmed, Eram S. Rao, Renuka Pathak, Geeta Trilok-Kumar

**Affiliations:** 1 Department of Food Technology, Bhaskaracharya College of Applied Sciences, University of Delhi, New Delhi, India;; 2 Department of Food and Nutrition and Food Technology, Institute of Home Economics, University of Delhi, New Delhi, India;; 3 Trivedi School of Biosciences, Ashoka University, Sonipat, India

**Keywords:** Catch-up growth, fetal programming, impaired glucose tolerance, insulin resistance, low birth weight, non-communicable diseases, type 2 diabetes

## Abstract

Insulin, secreted by pancreatic β-cells, regulates blood glucose levels. Insulin resistance (IR), characterized by diminished cellular responsiveness, increases insulin demand, imposing stress on β-cells. This leads to β-cell decompensation, which contributes to the development of type 2 diabetes (T2D). A growing body of evidence connects early-life exposures, such as low birth weight (LBW), to a heightened risk of developing non-communicable diseases (NCD) such as T2D in adulthood. This comprehensive review synthesizes findings from 15 observational studies identified through a PubMed search to explore the specific biological mechanisms that link LBW to IR and T2D. The paper critically examines several prominent hypotheses, theories, and models that propose how endogenous responses to intrauterine conditions initiate a programming that can lead to lasting structural and functional changes in key metabolic organs. In individuals with fetal programming, the combination of impaired insulin sensitivity and compensatory insulin action is considered a precursor to impaired glucose tolerance and T2D, particularly in those who underwent rapid catch-up growth during childhood. Regular monitoring of LBW individuals can enable early intervention, such as lifestyle changes, to reduce the risk of IR and T2D. However, improving the early environment by providing care for women of reproductive age, especially during pregnancy, is a cost-effective strategy to address LBW and curb the NCD pandemic.

## 1 INTRODUCTION

The pancreas is a lobular and elongated organ located obliquely beneath the upper and posterior abdominal wall [1]. It encompasses both exocrine and endocrine functions. The exocrine pancreas consists of acinar cells, which secrete digestive enzymes and bicarbonate, and a ductal system that drains the fluids into the intestine [2]. The endocrine pancreas is composed of five types of islet cells, which produce peptide hormones for maintaining glucose homeostasis: β- cells secrete insulin, α-cells produce glucagon, δ-cells secrete somatostatin, γ- or pancreatic polypeptide (PP cells) secrete pancreatic polypeptide, and ε-cells secrete ghrelin [3, 4]. Insulin, one of the endocrine peptide hormones, is secreted by the β-cells, which regulates blood glucose levels by enabling cellular glucose absorption, regulating carbohydrate, protein, and lipid metabolism, and stimulating cell division and maturation *via* mitogenic actions [5].

While greater than 5 mmol (90 mg/dL) of glucose concentrations in blood are needed to start insulin secretion, its biosynthesis is stimulated by fluctuations between 2 mmol (36 mg/dL) and 4 mmol (72 mg/dL) [6]. Food intake triggers glucose metabolism, which increases β-cell insulin synthesis and decreases glucagon secretion by α-cells to restore normal serum glucose levels [7]. Once secreted, insulin travels through the body, prompting hepatocytes to store glucose as glycogen. Adipocytes and skeletal muscle cells also absorb glucose, restoring blood glucose to normal levels [8].

Insulin resistance (IR) is defined as a decline in the cell's responsiveness to insulin in the blood. A person is said to be insulin resistant if higher circulating insulin levels are required to produce the same glucose-lowering response [9]. The resulting increased workload on the endocrine pancreas causes β-cell decompensation, an important contributory factor in the development of type 2 diabetes (T2D) [10-12]. Early-life exposures have been reported as possible risk factors for T2D and other cardiovascular diseases (CVD), in addition to heredity and poor lifestyle choices such as unhealthy eating habits and lack of physical activity [13, 14]. In this regard, several research studies indicate that low birth weight (LBW) at or near term, a surrogate measure of intra-uterine growth retardation (IUGR), may have a critical role in the emergence of non-communicable diseases (NCD) such as diabetes later in life [15-17]. IUGR has been shown to cause decreased muscle mass, decreased sensitivity to insulin in the muscles, and changes in the structure and function of beta cells. These alterations may eventually result in IR and dysfunction of beta cells [18-20].

Over the past three decades, scientists have proposed various hypotheses and models linking fetal nutritional restrictions to a higher risk of metabolic disorders later in life. Research suggests that intrauterine stress may modify early developmental pathways as an adaptive response, aiming to enhance survival and long-term health [21]. Barker hypothesized that poor prenatal nutrition triggers physiological reprogramming, making offspring adapted to undernutrition. The “thrifty phenotype” hypothesis suggests such individuals are more prone to metabolic disorders in nutrient-rich environments [20]. The theory of the Developmental Origins of Health and Disease (DOHaD) has been established based on this initial observation. Epigenetic variations in utero are emerging as key mediators of long-term developmental programming. Despite challenges in reproducibility, strong findings from animal models and human studies continue to shape this evolving field, with valuable insights surfacing amidst the publishing “noise” [22]. Expanding upon the thrifty phenotype hypothesis, the capacity-load model has been created to address the continuous association of early-life nutrition and growth patterns, as well as lifestyle variables later in life, with the risk of NCD, such as IR and diabetes. An alternative perspective posits that LBW may serve as a surrogate marker of IR established early in life, rather than acting as a direct causal factor [23]. In this context, in-utero IR impairs insulin’s function as a fetal growth factor, contributing to restricted growth. This line of reasoning is encapsulated in the ‘fetal insulin hypothesis’, which attributes such early dysfunction to both genetic predisposition and prenatal environmental influences [24].

While the link between LBW and later-life metabolic disease is well-established, the unique contribution of this review lies in its targeted exploration of the specific and often conflicting biological mechanisms that may explain the progression from LBW to IR and T2D.

## 2 SEARCH STRATEGY

A comprehensive literature search was conducted using the electronic database, PubMed, to identify relevant observational studies published in English between 1990 and 2025. The search employed various combinations of the following MeSH terms: ‘low birth weight’, ‘fetal programming’, ‘insulin resistance’, ‘impaired glucose tolerance’, ‘type 2 diabetes’, and ‘adults.’ The inclusion criteria were pre-defined to focus on observational studies that examined the association between LBW and the risk of IR and T2D in adulthood. Studies that investigated these associations exclusively in pediatric populations were excluded. The selection process is illustrated in Fig. (**1**). Out of a total of 5916 articles retrieved, 15 studies met all the eligibility criteria and were ultimately included in this review.

## 3 INSULIN SIGNALLING, INSULIN RESISTANCE, AND PROGRESSION TO TYPE 2 DIABETES IN LOW BIRTH WEIGHT ADULTS

Insulin works by attaching to a cell surface receptor and activating tyrosine kinase. Insulin receptors are found on the surface of almost all cells, but are highly expressed in skeletal muscle, liver, and adipose tissue, which are the main insulin target tissues [25].

Normal blood glucose levels vary from 5.0 to 6.1 mmol/L (90 to 110 mg/dL) but can increase to 14 to 17 mmol/L (250-300 mg/dL) after a meal [26]. During fasting, glucose is secreted into the bloodstream by the liver to maintain euglycemia and fuel glucose-consuming cells. Hepatic glucose production (HGP) breaks down hepatic glycogen (glycogenolysis) and synthesizes glucose (gluconeogenesis) from fatty acids and glycerol from adipose tissues [27]. Following food intake, pancreatic β-cells release insulin, which stimulates anabolism and decreases catabolism. During the metabolism of glucose, insulin enhances glucose absorption in insulin target tissues and boosts glycogen and lipid synthesis in these sites [28]. Insulin reduces HGP *via* suppressing gluconeogenic gene expression and lipid breakdown in adipose tissue. Additionally, insulin inhibits the release of glucagon from pancreatic α-cells and decreases appetite through the central nervous system [9]. The regulation of glucose homeostasis by the pancreas has been illustrated in Fig. (**2**) [29].

When a high level of insulin following a meal is unable to regulate this process due to IR, elevated glucose stays in the bloodstream for a longer duration, as in the case of T2D [26]. Individuals with IR and T2D commonly exhibit aberrant lipid metabolism, elevated circulatory concentration of lipids, and increased lipid accumulation in skeletal muscle and visceral tissue [30, 31]. Increased plasma lipid levels hinder insulin action, and increased free fatty acids (FFA) inhibit insulin-stimulated glucose absorption, whereas a reduction in lipid content increases insulin sensitivity [32]. It has been demonstrated in age and body mass index-matched controls that there is an inverse relationship between plasma FFA concentration and insulin sensitivity [33]. These findings indicate that obesity-related increased lipid content is the main contributor to IR and T2D.

While several theories aim to explain how increased lipid availability leads to IR [34], a compelling area of research connects these metabolic disruptions to early life factors. Specifically, a growing body of evidence, as discussed in this review, highlights the association between LBW and an increased risk of developing IR, laying the groundwork for the natural progression of T2D. A recent systematic review and meta-analysis including 135 studies involving adults found a link between birth weight and T2D, CVD, and hypertension; however, data on children and adolescents remains limited [17]. In this study, scientists discovered a J-shaped relationship between T2D and birth weight, with those weighing between 3.5 kg and 4 kg having the lowest risk. In a cohort of 12,525 middle-aged and older Brazilian adults, sex-specific associations were observed between LBW and T2D, as well as measures of glucose homeostasis. LBW was associated with higher T2D prevalence in women irrespective of maternal diabetes status, whereas in men, this association was evident only in the presence of maternal diabetes [35]. These sex-specific findings align with a Danish study of 2,23,099 adults, where LBW was more strongly associated with adult T2D risk in women than in men [36].

Endogenous responses to the poor intrauterine environment during a period of critical development appear to be the cause of the association of IR and LBW, leading to a programming that begins in the fetal period and continues throughout life [37]. IR and compensatory hyperinsulinemia have been suggested to contribute to chronic illnesses in persons with fetal programming [38]. Low insulin sensitivity and high secretory response have been found to be independent predictors of impaired glucose tolerance (IGT) and T2D in some follow-up studies [38-41]. Therefore, the combination of reduced insulin sensitivity and compensatory insulin action is thought to be a precursor to IGT and T2D [42].

In a study conducted on young adults belonging to the Helsinki Study of very low birth weight (VLBW) adults, reduced IR was compensated by enhanced insulin secretory response [43]. This prevented a rise in plasma glucose concentrations, resulting in most young adults staying normoglycemic. Another study reported a similar finding, indicating that adults in their mid-twenties born with VLBW had higher IR, although the prevalence of dysglycemia remained the same [41]. According to population-based studies that used administrative data to diagnose diabetes, adults born before 35 weeks of gestation had a 1.5 to 2-fold higher prevalence of T2D by the time they were 40-60 years old [44, 45]. Several other studies examining the association between LBW, IR, and T2D have reported similar findings [46-53].

Thus, IR is seen much earlier than the rise in fasting blood glucose level. Insulin is still able to maintain blood glucose levels within acceptable limits in individuals who gradually become more insulin-resistant. This can persist for years or even decades. Often, we focus on glucose as the issue, ignoring IR until it becomes insufficient to control blood glucose levels. This is when T2D eventually develops, maybe decades after the IR first started [54]. Table **1** displays original research studies that link LBW with IR and T2D during adulthood.

## 4 THEORIES AND HYPOTHESES ASSOCIATING LOW BIRTH WEIGHT WITH INSULIN RESISTANCE AND DIABETES

### 4.1 Fetal Metabolic Programming and the Role of Epigenetics

Barker and Hales introduced the concept of fetal metabolic programming in 1998, which later evolved into DOHaD theory. According to this theory, a poor intrauterine environment leads to fetal metabolic programming, which eventually results in adult metabolic disorders. Fetal metabolic programming causes lasting modifications to metabolic signaling pathways during growth and development, resulting in irreversible structural and functional changes [55-57]. Suboptimal intrauterine conditions have been linked to IUGR, LBW, and increased risk of metabolic syndrome during adulthood [58]. It has been proposed that a “thrifty phenotype” develops because of adaptive responses to a suboptimal fetal nutritional environment, which is characterized by a relative decrease in overall body growth as well as in the growth of organs such as pancreas and liver, while the growth of key organs such as brain and heart is differentially spared [20, 59].

Although highly unlikely to be encoded in the DNA sequence, the molecular mechanism(s) behind this phenomenon are mostly conjectural. Epigenetic disruption has surfaced as a leading contender for the environment-mediated molecular alterations underlying fetal programming [22]. Epigenetics refers to the inheritance of variation in the expression of genes without alterations to the sequence of DNA nucleotides. Epigenetic changes can be transferred stably during cell growth. These changes may be carried out by methylation of DNA, alterations to histone, chromatin remodeling, non-coding RNA, genomic imprinting *etc.* This mechanism controls the pre-transcriptional (heterochromatinization), transcriptional (chromatin activation, binding of the cis-acting component and the trans-acting factor), and post-transcriptional (translation of protein) levels of gene expression [60-63].

Epigenetic programming may have a significant impact on fetal metabolic programming through epigenetic alteration [64, 65]. The unfavorable intrauterine environment during fetal development is more likely to elicit epigenetic changes with no genetic modifications, and is very steadily passed down till adulthood [66], thereby increasing the susceptibility towards metabolic syndrome, such as IR during adulthood. Epigenetic programming has a sensitive window during the development of germ cells, embryo, and fetus [67].

#### 4.1.1 Epigenetic Regulations in Intra-uterine Growth Retardation

Epigenetic processes primarily include DNA methylation, modification of histone, and regulation of microRNA. DNA methylation is a highly robust and well-understood epigenetic pathway and involves introducing a methyl group to the fifth carbon of cytosine-guanine dinucleotides. Highly methylated DNA sequences, particularly promoters, often limit gene expression [68]. In light of the fact that epigenetic marks possess the ability to regulate gene expression, a significant factor in numerous diseases, it has been proposed that these marks act as a potential pathway by which in-utero exposure to an adverse environment results in the development of diseases such as obesity and T2D [69, 70].

##### 4.1.1.1 Human Studies

Despite scant human data, some studies have shed light on the potential epigenetic pathways that contribute to the epigenetic programming of obesity and T2D. The first research to show a connection between the prenatal environment and modified epigenetic profiles in the offspring was done on the children whose mothers were pregnant at the time of the ‘Dutch Hunger Winter’ in 1944-45 [71, 72]. During World War II, the scarcity of food in North Holland led to this catastrophic famine. Researchers first noticed that young males born to mothers pregnant during the famine had a higher risk of becoming obese [71]. Studies revealed that six decades later, in comparison to their unexposed, same-sex siblings, individuals exposed to the famine prenatally had less DNA methylation at the imprinted insulin-like growth factor-2 (IGF-2) gene region. As a result, it was determined that the stage of early pregnancy was crucial for exposure to famine [72, 73]. The same group of researchers analyzed methylation of DNA at 15 genomic sites with genes associated with growth, development, and metabolism of energy. Six of these genes showed variations in DNA methylation in adulthood based on intrauterine famine exposure [74]. In children of both genders, methylation alterations in the blood have been linked to genes associated with growth and an elevated risk of metabolic disorders [75].

##### 4.1.1.2 Animal studies

Animal studies have helped to demonstrate the impact of epigenetically induced fetal programming on disease risk by studying mammalian development in relevant organs and under controlled conditions. Studies in mice indicate that maternal malnutrition, stress, and micronutrient status might impact offspring physiology, leading to alterations in the methylation of critical metabolic or regulatory genes [76-80].

Several animal models have shown that DNA methylation has a significant impact on the phenotype of developing fetuses. A study found that feeding genetically obese Agouti mice a methylating cocktail containing B_12_, choline, folic acid, and betaine resulted in pups with a different coat color and decreased obesity, despite carrying the Agouti mutation. The methylation status of the Agouti gene's promoter region was a contributing factor [80]. In a similar study, scientists induced a methionine deficit in female sheep by restricting their intake of methionine, B_12_, and folate [81]. Fertilization of the sheep's ova was conducted *in vitro*, and the resulting blastocysts were administered to surrogate mothers who had normal methionine levels. The progeny, particularly males, displayed obesity and IR, with differential methylation at many genomic locations. In fetal programming, these models demonstrate the significance of 1C (methyl) metabolism during periconception.

### 4.2 Early Catch-up Growth

Catch-up growth refers to a period of increased growth velocity that occurs after a phase of growth restriction, aiming to restore the individual’s growth pattern toward the expected norms for preterm or term-born infants [82]. The early catch-up theory emphasizes the significance of malnutrition in the perinatal stage of life [83]. In a similar postnatal environment, in-utero modifications can enhance survival. If the postnatal environment differs significantly from the prenatal environment, the fetal “programs” fail to adapt, leading to the development of metabolic diseases [84]. This is also explained by the ‘predictive adaptive response’ hypothesis, which states that the fetus would adjust during gestation or in the early postnatal phase based on the “predicted” postnatal environment. Therefore, IUGR fetuses would predict a suboptimal nutritional environment postnatally [85]. Mismatch between predicted and actual outcomes would lead to disease manifestation [85, 86]. Recovery from intrauterine insults during childhood, also known as catch-up, results in faster accumulation of fat mass compared to muscle mass [87]. Very often, LBW children are overfed, which causes inadequate neuroendocrine circuit programming [17], epigenetic mechanisms, and cellular aging [88] that eventually result in fast catch-up growth and lifelong endocrine disturbances [88-90].

According to the Pune Children Study, LBW was linked to IR as early as 4 years old [91], and children who were LBW but gained weight during childhood were at risk for developing diabetes and CVD [92]. Replicable results have been obtained in populations with varying genetic backgrounds and dietary habits [59, 93-99]. According to these studies, those born small and who were thin during infancy but experienced rapid weight gain during childhood, resulting in an average or above average body mass index (BMI) by the time they were 11 years old, had an increased risk of developing T2D in adulthood. Patients with IGT or T2D typically have a low BMI during infancy, childhood adiposity rebound, and a rapid increase in BMI till adulthood [94]. A study in which catch-up growth occurred within the first year of life reported no effect on the derived insulin or glucose concentrations measured at 7 years of age [100]. Similarly, weight gain from birth to 18 months showed no impact on insulin levels at 9-12 years of age [101]. Other studies have also shown no rise in IR with catch-up growth by the end of the first year [102-104]. It is suggested that early catch-up growth after birth is a natural process to restore body size and does not influence insulin metabolism [103].

At the time of birth, IUGR infants have low levels of insulin, insulin-like growth factor-1 (IGF-1), and its binding protein- IGFBP-3 concentrations at birth, but high levels of growth hormone, IGFBP-1, and IGFBP-2. During the first trimester of postnatal life, the concentrations of these hormones return to normal [105, 106]. Tissues that have been chronically depleted of insulin and IGF-1 during fetal life may counteract the surge in the levels of these two hormones during this early postnatal catch-up growth period by developing IR as a metabolic defense against developing hypoglycemia [107]. This is consistent with findings from Caucasian and Asian populations where LBW neonates exhibit early markers of IR, characterized by elevated glycosylated haemoglobin and glycated serum protein levels [108, 109]. Thus, infants with IUGR who exhibit early and full growth recovery may have a higher risk of developing metabolic syndrome as adults.

#### 4.2.1 Role of Leptin during Catch-up Growth

The hormone leptin, which is primarily generated and released into the bloodstream by adipose tissue, is essential for the chronic regulation of insulin sensitivity, energy balance, and hunger [110]. Mature adipocytes are the primary source of leptin expression [111, 112], and plasma leptin concentrations are often correlated with the overall fat mass in individuals [113]. Leptin is not only a crucial adipokine but also a marker of the terminal differentiation of adipocytes [114]. It has been proposed that childhood nutrition and growth are associated with modified body composition, IR, and T2D, possibly due to altered programming of the processes regulating gene expression of leptin and its release from adipocytes [115].

Research shows that infants born with LBW may have high levels of leptin during catch-up growth, indicating adaptive leptin resistance that can persist into adulthood [51, 116]. Another hypothesis associates catch-up growth with adipocyte hypertrophy in male rats [117]. Adipocytes from LBW animals or hypertrophic adipocytes have a unique gene expression [118] and are strongly linked to high concentrations of leptin [119]. Adults born with LBW may have a high leptin to fat mass ratio because of malnutrition in the womb [120].

### 4.3 Capacity-load Model

The risk of NCD is increased by several factors related to adult lifestyle and environment, such as food, sedentary habits, stress, alcohol use, air pollution, smoking, and infections. Combined, these put the body under a “metabolic load,” making it more difficult for cells, organs, or tissues to maintain homoeostasis [121, 122]. Subsequently, it is considered that a group of characteristics known as “metabolic capacity”, which facilitates homeostasis, is necessary to withstand metabolic load [121, 122]. These characteristics emerge during early “critical windows,” indicating that growth trends and nutrient supply throughout the fetal period and infancy significantly shape them, in line with the thrifty phenotype theory [123].

The capacity-load model and the thrifty phenotype hypothesis differ primarily in that the former places a continuous model of NCD risk in lieu of the latter's emphasis on early malnutrition [123]. The dose-response relationships between early developmental variability and organ phenotype are highlighted by the capacity-load model [121, 122]. Numerous physiological characteristics, such as newborn lean mass, the number of nephrons in the kidney, airway size, and metabolic processes like insulin secretion, correlate fairly linearly with birth weight. These attributes are generally strengthened if lean mass is higher at birth, which translates to stronger long-term homeostatic capability. Although metabolic capacity is believed to continue from birth into adulthood, it gradually declines as people age. When homeostasis is compromised, pathophysiology can develop, which eventually leads to overt NCD such as T2D [123]. The NCD risk may be modelled based on metabolic load and capacity. Increasing load is expected to raise NCD risk in a dose-response manner while maintaining constant capacity. Similarly, reducing capacity is expected to increase risk in a dose-response manner while maintaining constant load. Individuals with decreased capacity and increased load are at the highest risk of developing NCD [121, 122].

### 4.4 Fetal Insulin Hypothesis

The fetal programming hypothesis posits that LBW in neonates is influenced by maternal factors. In contrast, Hattersley and Tooke introduced the fetal insulin hypothesis, which attributes the association between LBW and adult IR primarily to genetic determinants [24]. This hypothesis suggests that IR, present in utero and at birth, impairs the function of insulin and insulin-like peptides as growth-regulating hormones, thereby contributing to CVD observed in adulthood. LBW, IR, T2D, and coronary artery disease are proposed to stem from a common insulin-resistant fetal genotype, independent of environmental and maternal influences such as nutritional status and metabolic conditions [124].

Common yet unidentified polygenic factors contribute to IR both during fetal development and later in adult life. These genetic influences may result in two distinct phenotypes: a LBW, thin infant and an adult exhibiting IR, increased susceptibility to hypertension, atherosclerosis, and T2D, particularly when obesity is present. The observed correlation between fetal thinness and IR, even after adjusting for adult obesity, can be interpreted as two different manifestations stemming from the same underlying genetic mechanism. This concept provides a rationale for the inverse relationship between birthweight and glucose concentrations in oral glucose tolerance tests conducted five decades later [125]. The persistence of this association, despite the multitude of genetic and environmental factors influencing both fetal growth and glucose regulation, underscores its robustness.

Fig. (**3**) presents a conceptual framework of interconnected hypotheses that explain how exposures in early life may contribute to the onset of IR and T2D in adulthood.

## 5 ECONOMIC AND HEALTH POLICY IMPLICATIONS OF TYPE 2 DIABETES RISK AMONG ADULTS BORN WITH LOW BIRTH WEIGHT

Individuals born with LBW face an elevated risk of developing chronic conditions such as T2D, leading to significant long-term healthcare expenditures. The associated health challenges may contribute to reduced workforce participation and productivity, ultimately affecting economic growth. Governments and healthcare systems must prepare for increased demand in diabetes management, encompassing medication, hospitalizations, and preventive care [126].

To mitigate these risks, policies focused on enhancing maternal nutrition and prenatal care can play a crucial role in reducing the incidence of LBW, thereby decreasing future diabetes prevalence [127]. Early screening programs for IR in this population can facilitate timely intervention and more effective disease management. Additionally, nations with higher rates of LBW children should consider allocating greater resources to diabetes prevention and treatment, ensuring equitable access to healthcare [128].

## 6 COMBATING INSULIN RESISTANCE TO PREVENT THE LATER ONSET OF TYPE 2 DIABETES

To reduce cardiometabolic risks, including T2D, in individuals born with LBW, it is important to promote a healthy periconceptional and antenatal environment, as well as promote sound catch-up growth and prevent unnecessary fat and weight gain during infancy and childhood. Early detection of cardiometabolic abnormalities in LBW children and adolescents who are overweight and obese is crucial. Patients and families should be informed of their increased and early risk of T2D and CVD compared to their normal birth weight (NBW) peers, as well as efficient ways to reduce these risks [129]. Lifestyle modifications such as increasing physical activity and introducing dietary restrictions can significantly lower the risk of IR and even reverse it once it has occurred [54]. It is simple to take charge of our lives and change them for the better, and the best time to instill healthy lifestyle practices is during childhood. Childhood is an impressionable and habit-forming phase, and thus, the right time to introduce physical activity and a healthy dietary habit.

### 6.1 The Role of Physical Activity

In healthy and insulin-resistant individuals, physical activity raises insulin sensitivity by increasing nitric oxide synthesis [130], reducing vascular inflammation mediators [131], increasing high-density lipoprotein (HDL) cholesterol [132], and improving peripheral vasculature control [133]. A study conducted on children reported enhanced glucose tolerance and insulin sensitivity by maintaining a healthy weight and participating in sports [59]. Two studies involving middle-aged non-diabetic participants with a high risk of diabetes found a reduction in the diabetes incidence by 58% through a lifestyle-modification regimen combined with regular physical exercise [134, 135]. It has been established that the volume and intensity of an exercise program affect insulin sensitivity in distinct ways. A study found that exercise programs with around 170 minutes per week enhanced insulin sensitivity more than those with around 115 minutes per week [136]. Another study indicated that high-intensity physical activity has the greatest impact on lowering insulin levels [137]. The American College of Sports Medicine recommends 30 to 60 minutes of aerobic exercise with moderate-intensity five or more days a week, or three or more 20 to 60-minute high-intensity exercise sessions [138]. Combining resistance training with aerobic exercise may be more beneficial for managing blood glucose levels compared to either alone [139, 140].

### 6.2 Dietary Intervention

To fully understand the impact of food on blood insulin, it is important to determine the influence of each macronutrient. Dietary protein has a modest insulin impact (about twice as much as fasting levels, depending on blood glucose level). On the other hand, carbohydrates can cause a massive rise in insulin, more than ten times, and their height and length can vary greatly based on the kind of carbohydrate and the insulin sensitivity of the individual. Dietary fat has no impact on insulin whatsoever [141]. Therefore, a diet that reduces insulin spikers (refined carbohydrates) and enhances insulin dampeners (unrefined fat and protein) should increase insulin sensitivity.

#### 6.2.1 Carbohydrate

Since the 1990s, a strong body of clinical research has demonstrated that carbohydrate restriction reduces IR [54]. In a study, over a two-year period, participants were randomized to one of the three diets: non-calorie-restricted low-carb, calorie-restricted low-fat, or calorie-restricted moderate-fat. The non-calorie-restricted low-carb diet resulted in significant weight reduction while simultaneously improving insulin sensitivity and decreasing resistance [142]. Several meta-analyses including thousands of participants have consistently shown that a diet restricted in carbohydrates and unrestricted in calories may reduce insulin levels as much and many a times more than a diet restricted in carbohydrates and calories [143, 144]. In addition to the quantity of carbohydrates, their quality also matters. Assessing a carbohydrate's glycemic load (GL) is a helpful method to determine whether it is “good” or “bad.” GL is a figure that indicates how much a certain carbohydrate food will increase the blood glucose level after it is consumed. Pulses and legumes; cashews and peanuts; certain whole grain breads; millets; fruits; and vegetables are examples of low-GL foods and are also a good source of soluble fiber that are known to improve insulin sensitivity [145, 146].

#### 6.2.2 Protein

The impact of the protein intake on the insulin spike depends on the need of body for gluconeogenesis, *i.e.*, the biosynthesis of glucose in the liver from non-carbohydrate substrates when dietary glucose is not enough. Consuming protein with a high-carbohydrate diet causes a significant increase in insulin levels. However, a person who consumes very few carbohydrates has minimal to zero insulin response to protein [147].

#### 6.2.3 Fat

Low-carbohydrate diets, besides often being high in animal protein, are also often high in animal fat, which is usually a mixture of monounsaturated fatty acids (MUFA), polyunsaturated fatty acids (PUFA), and saturated fatty acids (SFA) [54]. Thus, contrary to a general belief, animal fat is not purely SFA, and SFA are not as bad as they are thought to be. Multiple meta-analyses have found no relation between SFA consumption and CVD [148-150]. A Dutch study on adult males and females, rather associated a lower risk of CVD with SFA consumption [151]. The risks associated with avoiding SFA include what we may substitute it with [54]. Replacing saturated fats, such as ghee, butter, *etc.*, with PUFA, such as corn oil, soybean oil, safflower oil, or rapeseed oil, may potentially cause greater damage [152-154]. Fat does matter, although not in the way one would assume. Ceramide is the form of fat that really matters [155]. It is a type of fat produced by our cells and is not something we should be concerned about in our diet. Inflammation activates ceramide synthesis, which has been linked to a reduction in insulin sensitivity. Importantly, no rise in ceramide levels is observed in the tissues of individuals following a fat-liberal, carbohydrate-restricted diet [156].

Thus, a carbohydrate-restricted diet plays an important role in controlling the impact of the other macronutrients- proteins and fats on insulin sensitivity. A low-carbohydrate diet can alleviate other factors that cause IR, including inflammation and oxidative stress [157, 158]. Therefore, carbohydrate restriction effectively addresses several reasons for IR.

## 7 STUDY LIMITATIONS

This review summarizes current evidence linking LBW to IR and T2D, but several limitations persist. Definitions of LBW and methodologies used to assess IR and diagnose T2D vary substantially, limiting comparability. Most data come from observational studies that cannot establish causality due to confounding factors, including genetic, environmental, and lifestyle influences. There is a notable lack of targeted intervention studies to reduce LBW-associated metabolic risks, restricting practical applications. Additionally, genetic diversity, regional differences in diet, and access to healthcare mean that findings may not generalize across all populations.

## CONCLUSION

Several hypotheses and models have demonstrated that LBW increases the risk of metabolic disorders, including IR, which may progress to T2D as age advances. It is interesting to note that IR appears well before aberrant glucose levels are observed. Regular monitoring of the LBW individuals will facilitate the identification of any cardiometabolic anomalies at an early stage, allowing for timely intervention, such as lifestyle modifications, to mitigate the risk of diabetes and impaired glucose tolerance in this group. Improving the early environment may be a more cost-effective way to prevent the NCD pandemic than focusing solely on lifestyle modifications made later in life. LBW is an avoidable public health issue and can be controlled by adequate nutritional, medical, and educational interventions for the women of reproductive age, especially pregnant mothers.

## AUTHORS’ CONTRIBUTIONS

The authors confirm their contribution to the paper as follows: study conception and design: SA, ESR, RP, and GTK; data collection: SA; Data Interpretation: SA and RP; methodology: SA, ESR, RP, and GTK; investigation: SA; drafting of the manuscript: SA. All authors reviewed the results and approved the final version of the manuscript.

## Figures and Tables

**Fig. (1):**
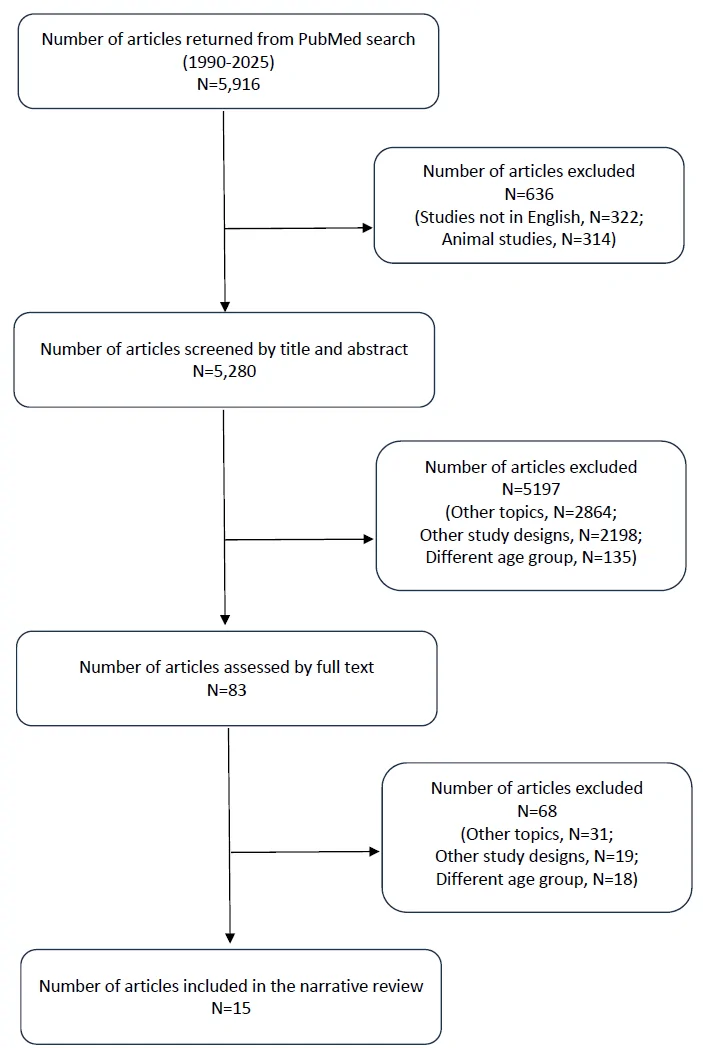
Flowchart of the article selection process for the review.

**Fig. (2):**
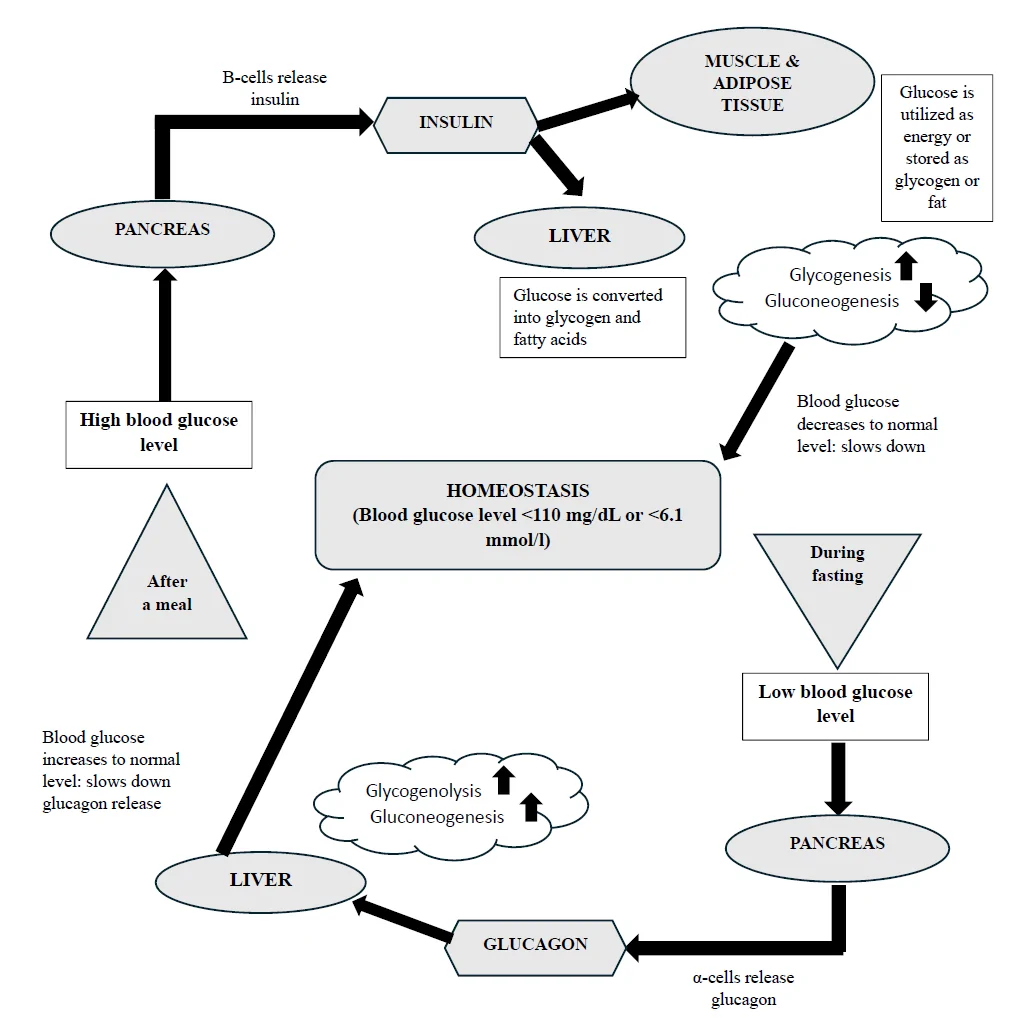
Regulation of glucose homeostasis by pancreas. (Available online under the terms of the Creative Commons Attribution Non-Commercial License 4.0.[29].

**Fig. (3):**
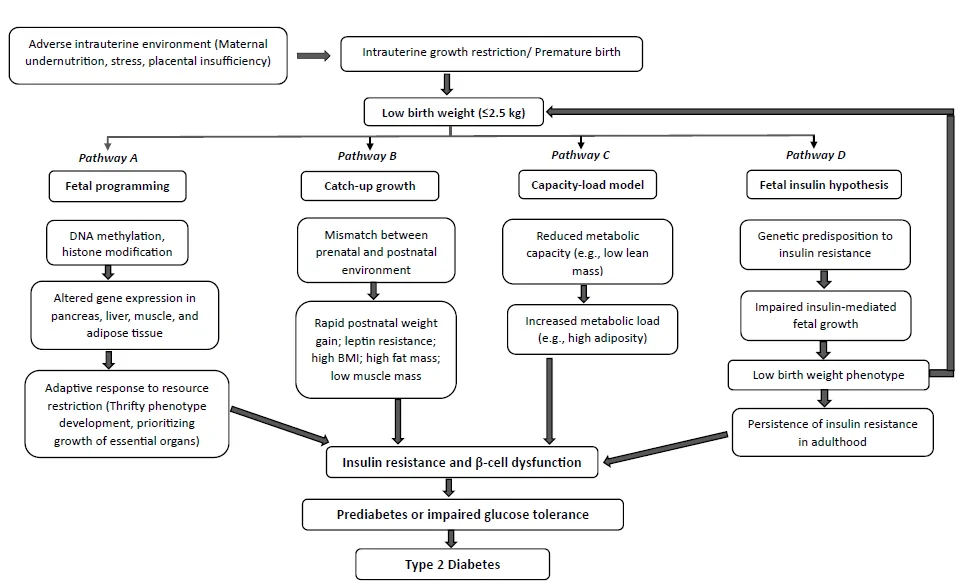
Integrated mechanisms of fetal programming: A conceptual flowchart illustrating the interconnected hypotheses linking early-life influences to adult metabolic disease.

**Table 1:** Original studies associating low birth weight with reduced insulin sensitivity and type 2 diabetes in adults^1^.

**S.No.**	**Author and Reference**	**Study Design**	**Location**	**No. of Participants**	**Tests Conducted**	**Conclusion**
1	Yarmolinsky *et al.* (2016) [35]	Cohort study	Brazil	15,105 volunteer employees, aged 35 to 74 years at baseline (2008-2010), from universities or research institutions.	OGTT measured glucose at baseline and 120 minutes, and HbA1c at baseline only.	• Low birth weight was linked to higher diabetes prevalence in women regardless of maternal diabetes, but in men only when maternal diabetes was present.
2	Rotteveel *et al.*, 2008 [40]	Cross-sectional	Denmark	29 adults born preterm AGA; 28 adults born preterm SGA; 30 adults born at term not SGA (controls).	The hyperinsulinemic euglycemic clamp technique was conducted to measure insulin sensitivity.	• Young adults who were born preterm exhibited reduced insulin sensitivity and elevated blood pressure compared to their full-term counterparts. • Gain in weight and height in childhood is was linked to reduced insulin sensitivity and elevated blood pressure in adulthood.
3	Hovi *et al.*, 2007 [41]	Cross-sectional	USA	163 adults with VLBW; 169 adults born at term and NBW.	OGTT measured glucose and insulin at baseline and 120 minutes	Young adults who were born with a VLBW showed greater insulin resistance, glucose intolerance, and elevated blood pressure compared to term-born non-SGA adults.
4	Kajantie *et al.*, 2015 [43]	Longitudinal birth cohort study	Finland	107 adults born with VLBW; 100 controls born at term, not SGA	14-sample IVGTT	• Adults born preterm with VLBW exhibited lower insulin sensitivity than those born at term with a comparable body size. • In young adulthood, this was compensated for by increased insulin secretion.
5	Kaijser *et al.*, 2009 [44]	Cross-sectional follow-up of a cohort	Sweden	2,931 participants were born at <37 weeks of gestation and 2,176 had a birth weight <2.5 g	Diabetes diagnoses were obtained from the Hospital Discharge Register.	The link between LBW and diabetes was attributed to factors related to both impaired fetal growth and a shorter gestational period.
6	Lawlor *et al.*, 2006 [45]	Cohort study	UK	A birth cohort of 5,793 individuals born in Aberdeen, Scotland, between 1950 and 1956 who completed a questionnaire in 2000.	Diabetes was defined based on self-report in a questionnaire.	LBW and preterm birth were linked to a higher risk of diabetes
7	Lithell *et al.*, 1996 [46]	Cohort study	Sweden	Birth records were traced for 1,333 of 2,322 men born in 1920-1924 and living in Uppsala in 1970.	Six sample and five sample IVGTT was conducted to assess glucose and insulin respectively.	• Findings confirmed the association between reduced fetal growth and an increased risk of diabetes, particularly linked to thinness at birth. • This relationship appears to be mediated by insulin resistance rather than impaired β-cell function and is influenced by obesity in adulthood.
8	Kaseva *et al.*, 2024 [47]	Cross-sectional study	Finland	74 VLBW and 70 same-sex sibling controls participated in the study.	OGTT was conducted to assess glucose and insulin at baseline and 120 minutes.	At young adulthood, individuals born with VLBW had a 3.4-fold higher odds of impaired glucose regulation compared to their same-sex term-born siblings.
9	Ronn *et al.*, 2019 [48]	Cross-sectional study	Greenland	1,429 participants were included from two population-based surveys.	Plasma glucose and serum insulin were measured using the OGTT at baseline and 120 minutes.	• An inverse relationship was observed between birth weight and both hepatic and peripheral insulin resistance, which was independent of adult adiposity. • LBW may play a role in the development of glucose intolerance among adult Inuit populations in Greenland.
10	Branda *et al.*, 2022 [53]	Cross-sectional study	Brazil	2634 participants from the ELSA-Brasil study.	Baseline samples of glucose and insulin were collected	• The observed association between HOMA-β and low LBW indicates that a decline in insulin secretion may occur in healthy middle-aged adults. • LBW may have a relatively reduced β-cell mass, which could contribute to an increased risk of glucose metabolism disturbances.
11	Yokoyama *et al.*, 2020 [49]	Cross-sectional study	Japan	1,135 men and women were part of this study.	OGTT was performed at 0, 60, and 120 minutes to estimate glucose and insulin.	In a Japanese population, LBW was linked to an elevated risk of T2D mellitus, particularly among overweight and obese individuals.
12	Pilgard *et al.*, 2010 [50]	Cross-sectional study	Denmark	4,644 participants from the Inter99 Danish intervention study were included.	OGTT was performed at 0, 30, and 120 minutes to measure glucose and insulin.	The association between birth weight and the risk of T2D was influenced by both β-cell function and insulin sensitivity.
13	Jornayvaz *et al.*, 2016 [51]	Cross-sectional population-based study	Switzerland	This study was conducted on 1088 men and 1458 women.	Fasting plasma glucose and insulin were estimated.	Middle-aged individuals born with LBW had a greater prevalence of obesity and diabetes, as well as increased leptin levels and a higher leptin-to-fat mass ratio, compared to those born with normal weight.
14	Phillips *et al.*, 2005 [93]	Cohort study	UK	768 men and 714 women from the Hertfordshire birth cohort participated in this study.	OGTT measured glucose and insulin at 0, 30, and 120 minutes.	The factors contributing to LBW or infant weight and impaired glucose tolerance in adulthood remained largely unchanged and continued to affect this cohort.
15	Bhargava *et al.*, 2004 [94]	Prospective population-based study	India	1492 adults from an Indian birth cohort participated in this study.	OGTT measured glucose and insulin at 0, 30, and 120 minutes.	• Thinness in infancy was linked to an increased risk of impaired glucose tolerance or diabetes in young adulthood. • Additionally, moving into higher body mass index categories after the age of two was also associated with these conditions.

^1^ SGA, small for gestational age; AGA, appropriate for gestational age; VLBW, very low birth weight; LBW, low birth weight; OGTT, oral glucose tolerance test; IVGTT, intra-venous glucose tolerance test; T2D, type 2 diabetes.

## LIST OF ABBREVIATIONS

IR: Insulin Resistance
T2D: Type 2 Diabetes
CVD: Cardiovascular Disease
LBW: Low Birth Weight
IUGR: Intra-uterine Growth Retardation
DOHaD: Developmental Origins of Health and Disease
NCD: Non-communicable Disease
HGP: Hepatic Glucose Production
FFA: Free Fatty Acids
IGT: Impaired Glucose Tolerance
VLBW: Very Low Birth Weight
IGF-2: Insulin-like Growth Factor-2
IGF-1: Insulin-like Growth Factor-1
BMI: Body Mass Index
NBW: Normal Birth Weight
HDL: High-density Lipoprotein
GL: Glycemic Load
MUFA: Monounsaturated Fatty Acids
PUFA: Polyunsaturated Fatty Acids
SFA: Saturated Fatty Acids
